# Public Opinion About COVID-19 on a Microblog Platform in China: Topic Modeling and Multidimensional Sentiment Analysis of Social Media

**DOI:** 10.2196/47508

**Published:** 2024-01-31

**Authors:** Feipeng Guo, Zixiang Liu, Qibei Lu, Shaobo Ji, Chen Zhang

**Affiliations:** 1 Modern Business Research Center Zhejiang Gongshang University Hangzhou China; 2 School of Management and E-Business Zhejiang Gongshang University Hangzhou China; 3 School of International Business Zhejiang International Studies University Hangzhou China; 4 Sprott School of Business Carleton University Ottawa, ON Canada; 5 General Manager's Office Hangzhou Gaojin Technology Co, Ltd Hangzhou China

**Keywords:** COVID-19, social media public opinion, microblog, sentiment analysis, topic modeling

## Abstract

**Background:**

The COVID-19 pandemic raised wide concern from all walks of life globally. Social media platforms became an important channel for information dissemination and an effective medium for public sentiment transmission during the COVID-19 pandemic.

**Objective:**

Mining and analyzing social media text information can not only reflect the changes in public sentiment characteristics during the COVID-19 pandemic but also help the government understand the trends in public opinion and reasonably control public opinion.

**Methods:**

First, this study collected microblog comments related to the COVID-19 pandemic as a data set. Second, sentiment analysis was carried out based on the topic modeling method combining latent Dirichlet allocation (LDA) and Bidirectional Encoder Representations from Transformers (BERT). Finally, a machine learning logistic regression (ML-LR) model combined with a sparse matrix was proposed to explore the evolutionary trend in public opinion on social media and verify the high accuracy of the model.

**Results:**

The experimental results show that, in different stages, the characteristics of public emotion are different, and the overall trend is from negative to positive.

**Conclusions:**

The proposed method can effectively reflect the characteristics of the different times and space of public opinion. The results provide theoretical support and practical reference in response to public health and safety events.

## Introduction

Due to the global influence of COVID-19, it has recently become a research hot spot in many fields [1]. Public opinion on social media refers to the public opinion expressed on social media software as the communication platform and social issues as the main topic, including public cognition and attitude [2]. Public opinion generated by public security and health emergencies such as infectious diseases spreads rapidly and is of an explosive nature [3]. Reasonable control of public opinion played an important role in curbing public panic during the COVID-19 pandemic [4].

In recent years, social media platforms have gradually become the main information dissemination medium for the public [5]. The use of social media to measure public attention has also been gradually applied to the research of acute infectious diseases, such as influenza A (H1N1) [6]. Ahmad et al [7] demonstrated a correlation between the daily number of COVID-19–related tweets and the daily number of COVID-19 cases and deaths in Iran and Turkey. Public knowledge was limited due to a lack of information about, inexperience with, and poor awareness of the nature of the virus. Text-based data on social media platforms contain rich information about public opinion and sentiment [8]. As of September 2022, the number of monthly active users of microblogging platforms reached 584 million, with mobile use accounting for 95%, and the number of daily active users reached 253 million. Although social media platforms disseminate information, they also present changes in public emotions [9,10]. Text-based data on social media provide accurate geolocations, rich emotional information, and distinct topics; the different topic information within social media text data is an important data source for public opinion research [11].

Most traditional spatiotemporal studies of public opinion use the administrative region as the analysis unit or the coordinates of users' blogs or comments to analyze the geographic density [12]. Box-Steffensmeier and Moses [13] explored the level of sentiment and severity of the pandemic in each state in the United States during the COVID-19 pandemic; however, they used only the administrative region as the unit of analysis, and the scope of analysis was limited. Liu et al [14] conducted spatiotemporal analysis of the evolution of public opinion during the COVID-19 pandemic based on microblog data and visualized public sentiment and the number of microblogs in provinces and cities across the country. Although the research scope was expanded to the whole country, the selected spatial units were relatively concentrated and were not able to reflect the differences in public opinion at different scales. The spread of the pandemic has obvious regional differences between cities. Liu and Liu [15] used the GeoSEn geoparser to obtain geographic location information from text data and converted it into coordinates for emotional spatial analysis, but the monitoring location was not accurate, which meant the analytic results deviated from the spatial standard. In particular, the results from administrative units at different levels are quite different, while there is a small number of microblogs with user location coordinates, which leads to further errors in the results. In addition, the propagation of public opinion on social media is regionally correlated. The scope of public concern not only is limited to local areas but also includes information from other adjacent areas, which cannot be effectively mined by traditional public opinion analysis methods [16].

Social media data published by users contain rich geographic information, and many scholars have used social media to simulate and predict real events, such as predicting the spread of influenza, detecting earthquakes, and monitoring air quality. To monitor public opinion, Hu et al [17] proposed a model combining latent Dirichlet allocation (LDA) based on the document generation model and the k-means text classification algorithm based on genetic optimization; this combination can improve the accuracy of the clustering algorithm to monitor public opinion. Specifically, this method can identify and track topics related to public opinion on Twitter as well as mine the public’s emotional characteristics in different fields. Zhang et al [18] studied algorithms to monitor a public opinion network, compared and analyzed the advantages and disadvantages of different text classification and emotional tendency algorithms, and discussed future trends in algorithm development to monitor public panic during the COVID-19 pandemic in different geographical locations. Microblogs contain emotional information from social users. Researchers such as Jang et al [19] have analyzed microblog data to determine the spatial and temporal variations in emotions expressed by Twitter users in North America. They also analyzed values given to emotions and the relationships between the influencing factors, which highlighted the study area index and emotional score. In their study, the spatiotemporal variation in the public's attention to the pandemic and emotion were discussed. Storey and O'Leary [20] constructed a global sentiment map of Twitter users and analyzed the spatiotemporal characteristics of urban sentiment by calculating the sentiment information from social media text-based data. The risk of pandemic spread at multiple time nodes was evaluated by converting geographic information from text into coordinates for emotional spatial analysis. Alhashmi et al [21] proposed a sentiment classification network model combining part of a speech attention mechanism and long short-term memory network, which can fully mine the relationship between emotional polarity words and emotional target words of sentences and analyze the correlation between the spatial distribution of online public opinion and high-risk areas of the pandemic through spatiotemporal migration.

This study aimed to mine social media comments on COVID-19 topics and explore their spatiotemporal distribution characteristics. The specific research period was from July 2020 to June 2021. More than 100,000 comments from 12 People's Daily microblogs were obtained through the microblog application programming interface (API) interface, and 2000 samples were screened for feature extraction and sentiment analysis. On this basis, we proposed 3 stages of the pandemic covering the changes in public opinion. Meanwhile, through topic modeling, 12 core topics were identified to map the potential drivers of these changes. The purpose of this study was to help government and public health security departments understand the public's emotions and views on the COVID-19 pandemic, formulate more reasonable pandemic prevention policies, effectively respond to the COVID-19 pandemic, and maintain social stability.

The rest of the paper is structured as follows: The Methods section introduces the research ideas and key algorithms used in this study. The Results section extends the analysis of the experimental results. The Discussion section concludes the paper with contributions, limitations, and directions for future research.

## Methods

### Overview

**Figure 1:**
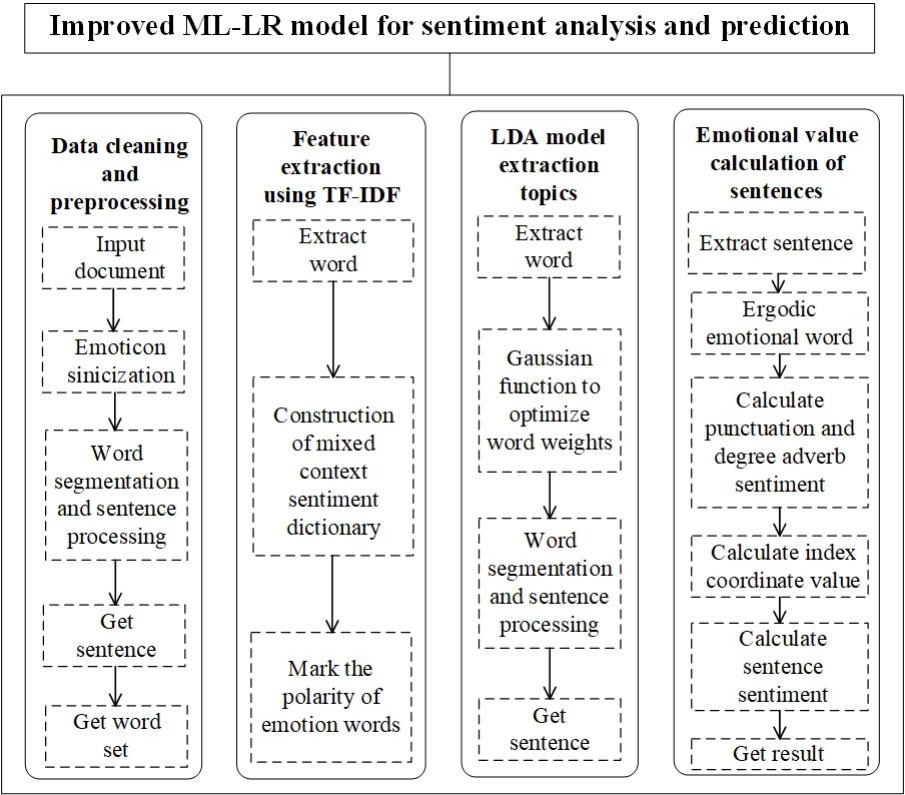
The system architecture of this study. LDA: latent Dirichlet allocation; ML-LR: machine learning logistic regression; TF-IDF: term frequency-inverse document frequency.

First, based on the API interface for the Sina microblog, data from COVID-19–related microblog comments from July 1, 2020, to June 1, 2021, which was in the middle of the COVID-19 pandemic, were obtained and preprocessed. Second, we considered the characteristic of low granularity as well as short words in the microblog comments [22]. Based on the SnowNLP Chinese sentiment vocabulary ontology, the Dalian University of Technology sentiment classification dictionary [23] is optimized to identify sentiment features, and a multidimensional analysis model of public opinion on social media during the pandemic was constructed from the perspective of spatiotemporal correlation. Third, according to the multiscale division method of public opinion, the term frequency-inverse document frequency (TF-IDF) method was used to explore the semantic features of public opinion. Fourth, according to the difference in public opinion data characteristics, a model combining LDA and Bidirectional Encoder Representations from Transformers (BERT) [24] was used to classify the topics. The number of topics was determined according to minimum perplexity, and the topic weight was established using chi-square test results. Finally, a machine learning logistic regression (ML-LR) sparse matrix fusion model was used to analyze the evolutionary trend in public opinion combined with the monthly pandemic notification information. The technical route and structure of this paper are shown in Figure 1.

### Data Extraction

This study collected data for topics related to COVID-19 in the monthly hot topic search of the People's Daily microblogs from July 2020 to June 2021, including the hot topic name, topic search volume, and date of the blog post with the most searched topic, and selected more than 100,000 comments from 12 microblogs. These 12 blogs have a high degree of heat (eg, likes, comments, and retweets), and the blog topics represent the hot events of the corresponding timeline, which can be regarded as a microcosm of the different development stages of the COVID-19 pandemic in China. The specific data from these 12 microblogs are shown in Table 1. Based on the ArcGIS interface of the geocoding package Geocoder, the geographic latitude and longitude of the check-in location coordinate system were then obtained, and the IP address geographic location information of the user was calculated. Through the Requests library of the Python language, the data interface was requested and parsed, and 2000 comments were screened for sentiment analysis. An example of the crawled microblog data is shown in Table 2.

**Table 1:** Data information statistics of 12 representative blogs about the COVID-19 pandemic in China from July 2020 to June 2021.

Topic	Date	Likes, n	Comments, n	Forwards, n
127 new cases were confirmed in 31 provinces	July 31, 2020	113,000	11,000	1453
Three new imported cases were reported in Wuhan	August 2, 2020	62,000	4987	1015
Yunnan released details of a new asymptomatic case	September 1, 2020	56,000	2973	811
Qingdao has collected 1.03 million samples for nucleic acid testing	October 12, 2020	61,000	3257	1580
Wu Zunyou said cold chain imports or become the source of the pandemic in China	November 11, 2020	30,000	2464	3072
A COVID-19 vaccine has been approved for marketing in China	December 31, 2020	720,000	40,000	93,000
Zhong Nanshan thanks you for not going home during the Spring Festival	January 25, 2021	113,000	8871	6025
China's medium and high-risk areas are cleared today	February 22, 2021	8935	1093	1498
Zhong Nanshan says there are dangers in not getting vaccinated in time	March 31, 2021	453,000	37,000	13,000
Cluster infection occurred among Chinese students	April 30, 2021	6697	673	235
The confirmed cases in Liwan, Guangzhou were infected with Indian variant strain	May 23, 2021	270,000	10,000	5009
Zhong Nanshan said the concept of close connection should be updated	June 26, 2021	101,000	4134	15,000

**Table 2:** Sample comment information about the COVID-19 pandemic in China crawled from a microblog platform from July 2020 to June 2021.

ID	Date	Review	IP
User 1	October 15, 2020	It is hoped that the test reagents can be distributed to every city in the country, whether developed or backward. The more backward provinces are the bigger the crisis, the news is closed, and the awareness of prevention is weak	Jilin Province
User 2	January 24, 2021	The most frightening thing is that the virus is still in its initial stage, and how it will mutate in the future is uncertain. What if it becomes more deadly	Guangdong Province
User 3	April 8, 2021	We call on the government to strictly control the price of masks and crack down on the bad behavior of price gouging by bad businesses in special periods	Beijing City
User 4	June 17, 2021	With such attention and rapid action and scientific prevention and treatment, the country will be able to effectively control and defeat the COVID-19 pandemic	Sichuan Province

### Data Preprocessing

Issues with the crawled text data included missing column values, numerical anomalies, and special symbols, which need to be supplemented and filtered. First, the Jieba word segmentation tool was used to process the text segmentation, and a custom dictionary was established for the high-frequency words and proper nouns related to the pandemic. Second, the Trie tree structure was used for efficient word graph scanning, and a stop word list was established to filter out the noisy data from the text. Third, the text information was converted into vectors (ie, feature engineering transformation). The model can only input numbers (like vectors), not text; therefore, before running the model, any signal needed to be converted into a digital signal (eg, number, vector, matrix, tensor) that can be recognized by the model. The regular word segmentation result was used as the input for the model. The data preprocessing results are shown in Table 3.

**Table 3:** Comparison of a sample of annotated texts about COVID-19 in China before and after data preprocessing from July 2020 to June 2021.

Number	Review	
	Before pretreatment	After pretreatment
1	Now the pandemic is spreading, the whole network selling masks are doubling the price! Really no conscience!	[pandemic], [spread], [sell], [mask], [price rise], [no], [conscience]
2	We should praise the medical staff and scientific researchers who are still struggling in the front line!	[for], [struggle], [medical care], [scientific research], [personnel], [praise]
3	You don't need to panic, but you do need to be vigilant. If you notice suspicious symptoms, go to the hospital.	[don't], [in], [but], [vigilant], [if], [notice], [symptoms], [to], [hospital], [medical]

The specific text data preprocessing process included (1) removing duplicate data, (2) removing useless characters, (3) using Jieba word segmentation, (4) removing stop words, (5) removing low-frequency words, (6) removing any tags such as HTML, and (7) using the decision tree method to supplement the missing column values and integrate the data.

### Sentiment Classification

To reflect the characteristics of public opinion, a method based on dictionary and thesaurus matching was used. The Dalian University of Technology sentiment dictionary was used to scan the strings in the dictionary one by one and provide auxiliary annotations for sentiment classification. This method combines the characteristics of the topic and Chinese grammar and expands the original dictionary from the dimensions of “word,” “part of speech,” “word sense number,” “sentiment classification,” “intensity,” and “polarity.”

The basic dictionary selected for this paper was the emotional vocabulary library of the Dalian University of Technology, which divides emotions into 7 categories (“anger,” “disgust,” “fear,” “sadness,” “surprise,” “good,” “happy”) and 21 subcategories. The initial emotional intensity was set to 5 levels (1, 3, 5, 7, 9), which is more detailed than other dictionaries. To facilitate sentiment calculation by a computer, we divided the polarity of sentiment words into 2 categories: positive (1) and negative (0). The formula for word sentiment is given in Equation 1:

*s*(*w*)=*v*(*w*)*p*(*w*) **(1)**

where *s*(*w*) represents the sentiment value of the term, *v*(*w*) is the sentiment intensity of the word, and *p*(*w*) denotes the sentiment polarity of the word.

First, we needed to calculate the TF-IDF values of the words in the text to obtain the feature matrix. The TF-IDF value 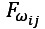 of the feature item *w*_ij_ was calculated as shown in Equations 2-5:



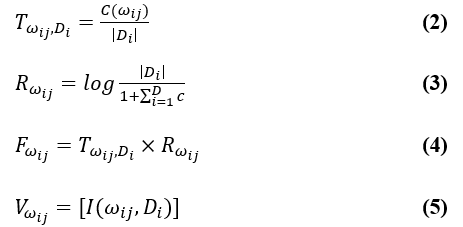



Here, *w*_ij_ represents the *j*_th_ word appearing in the microblog text-based data from the *i*_th_ day. *C*(*w*_ij_) is the number of occurrences of the term *w*_ij_. *D* is the total number of documents in the daily microblog text-based data |*D*_i_| for the number of words in a document *D*_i_. 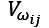 denotes the bag-of-words vector of the document *D*_i_. The function *I*(*w*_ij_,*D*_i_) takes the value 1 or 0, where 1 means that the document *D*_i_ contains the word *w*_ij_; otherwise, it takes the value 0. The part-of-speech and sentiment classification of the words are shown in Table 4.

**Table 4:** Part-of-speech and sentiment classification of words in the text of a sample of microblog comments with the topic of COVID-19 pandemic behavior in China from July 2020 to June 2021.

Word	Part of speech	Sentiment classification	Intensity	Polarity	Auxiliary classification
Anger	Adjective	NAU^a^	7	–1	NaN^b^
Fear	Adjective	NI^c^	5	–1	NG^d^
Grief	Verb	NB^e^	7	–1	NJ^f^
Surprise	Adjective	PC^g^	6	1	NaN
Hope	Noun	PD^h^	4	1	PH^i^
Pleasure	Verb	PA^j^	4	1	PE^k^

^a^NAU: anger.

^b^NaN: null value.

^c^NI: panic.

^d^NG: shame.

^e^NB: sadness.

^f^NJ: disappointment.

^g^PC: surprise.

^h^PD: respect.

^i^PH: praise.

^j^PA: happiness.

^k^PE: peace of mind.

### Feature Extraction

This study used a topical term extraction method based on the combination of LDA and BERT. First, the review text was segmented into words, and the word vector was generated based on the hybrid pretrained model. The k-means clustering method was then used to cluster the word vectors, and the words with higher word frequencies were selected as representative words in each class. At the same time, other words in the same class were replaced by representative words to reduce the amount of input data. Finally, the biterm topic model (BTM) for short text was used to extract the topics.

The BTM is aimed at the characteristics of short text and extracts more informative topics using co-occurrence patterns of word pairs in the whole corpus. The modeling process of the BTM generates a corpus of word pairs. Model training and model parameter inference are then performed based on the generated corpus. Finally, the topic distribution and word distribution on the corpus are obtained. If we suppose there are *M* feature words, |*B*| word pairs, and *K* topics in the corpus, then the corpus-level topic distribution is denoted by *θ*, the distribution of topic *K* is denoted by *θ*_k_, and the word distribution is denoted by Ø, as shown in Equation 6.



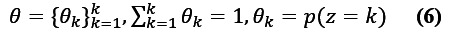



where *z* represents a topic extracted, *p*(*z*=*k*) is the probability of occurrence of topic *k*, and *p*(*z*=*k*) is the probability of occurrence of words under topic *k*.

Perplexity is widely used to measure the fitting effect of an LDA model. Perplexity is a probabilistic model evaluation metric that evaluates the predictive ability of a trained model on new unseen data. LDA perplexity is the perplexity when the model predicts new unseen text. After understanding the calculation of the statement probability, the perplexity of the statement *s*=*w*_1_, *w*_2_, *w*_3_, ..., *w*_n_ can be defined as seen in Equation 7:



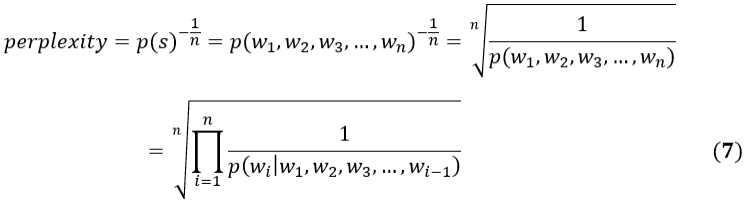



To calculate the perplexity of LDA, we needed to utilize the generation process of the model. Specifically, we needed to split the text data into training and test sets. The training set was used to build the LDA model, and the test set was used to calculate the perplexity of the model. In the test set, we treated each document as a sequence, taking one word at a time from the sequence and inferring the topic of that word based on the current model. Once the topic of the word was predicted, the model parameters were recalculated. In this process, we needed to calculate the perplexity of the text using the topic of the predicted word and the frequency of all the words.

### Sentiment Analysis

The microblog comment text was divided into positive and negative sentiment, and the value represented the probability that the text contained that sentiment. The values ranged from 0 to 1; closer to 1 tended to be positive, and closer to 0 tended to be negative. To improve the accuracy of sentiment prediction, the sentiment classification model needed to be retrained. The main steps are described in the following paragraphs.

First, the training method of the Bayesian model was used to train the sentiment classifier, and the classification method in the Bayesian classification was then used to predict the sentiment classification and test the accuracy of the model. Finally, the newly trained model was saved.

The daily microblog text set *D*={*d*_1_, *d*_2_, ..., *d*_m_}. The variable m represents the number of microblog crawls per day. The sentiment score 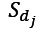 of each microblog was obtained using the newly trained sentiment analysis model. In the actual judgment, to make the visualization results more intuitive, the value of 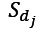 was lowered by 1. That is, when the return value was in the interval of –1 to 0, the sentiment probability value was negative; when the return value was between 0 and 1, the sentiment probability value was positive. The sentiment score *S*_D_ was calculated as shown in Equation 8:



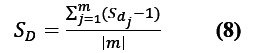



First, the feature words were inputted, and the sentiment polarity was classified as 1 (“positive”) or 0 (“negative”). Second, the TF-IDF calculation converted the words in the text into a word frequency matrix, and the matrix element *a*[*i*][*j*] represented the word frequency of *j* words in type *i* text. *MemoryError* was used to control the parameter. The transformer was then used to calculate the TF-IDF weight of each word. The first *fit_transform* was used to calculate the TF-IDF, and the second *fit_transform* was used to convert the text into a term frequency matrix to obtain all the words in the bag-of-words model. The TF-IDF matrix was then extracted. The element *w*[*i*][*j*] represented the TF-IDF weight of *j* word in the *i* type text, and the data were divided by combining the sparse matrix. Finally, the logistic regression classification method was used to calculate the model benchmark indicators. The binary classification ML-LR model is given in Equations 9 and 10:



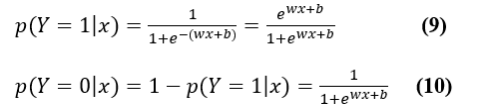



where *w* is the weight, *b* is the bias (*b* can be 0), and we can expand the weight and input vector a little, as shown in Equation 11:

*w*=(*w*^(1)^, *w*^(2)^, ..., *w*^(m)^, b)*^T^*, *X*=(*x*^(1)^, *x*^(2)^, ..., *x*^(m)^, 1)*^T^***(11)**

### Ethical Considerations

This study did not require ethics approval because all data collected were publicly available. There is no means within this paper or its supporting materials to establish the identification of users and their corresponding tweets.

## Results

### Analysis of the Change in Public Opinion Based on LDA Topic Modeling

**Figure 2:**
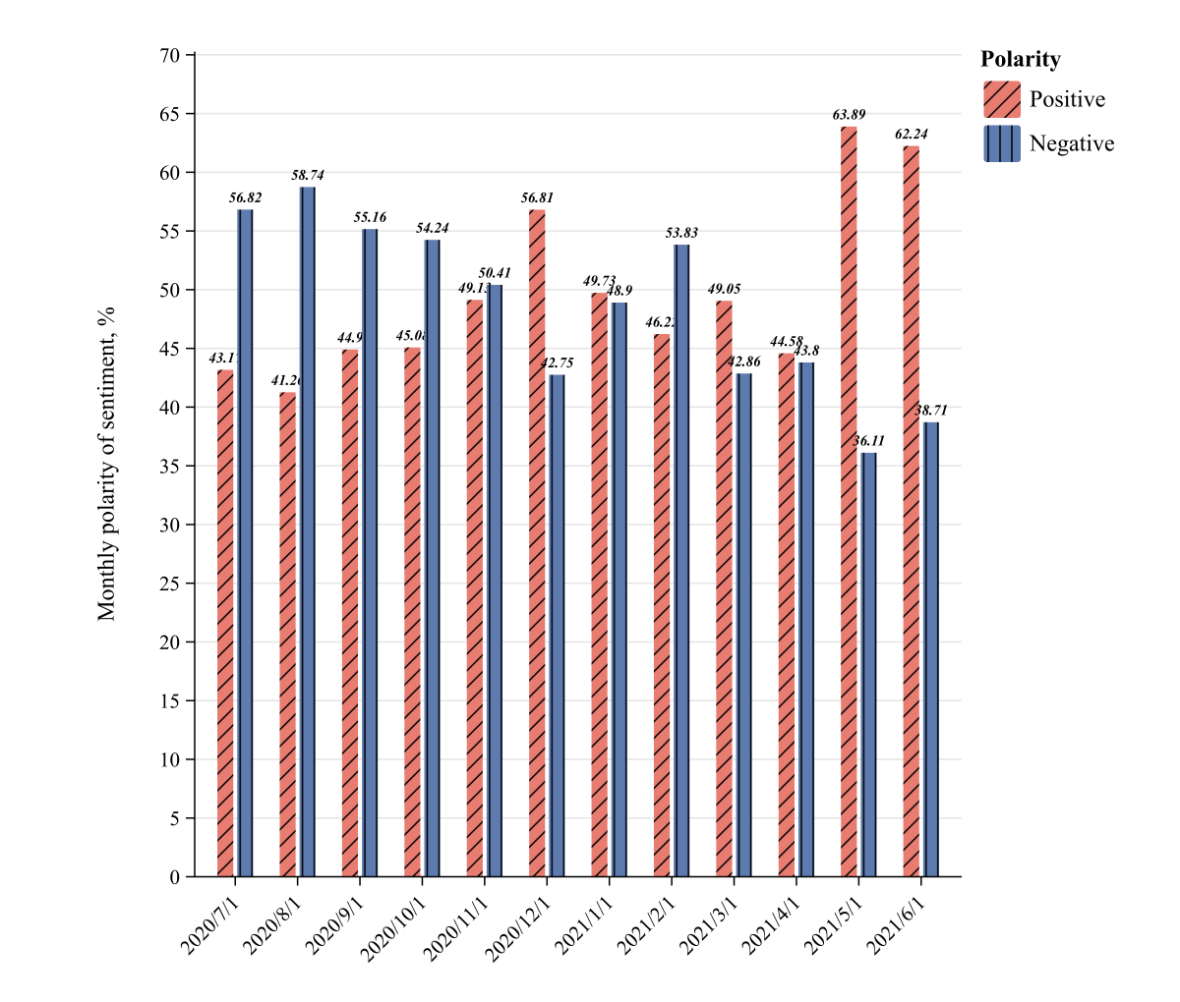
Monthly distribution of the sentiment polarity in microblog comments about the COVID-19 pandemic in China from July 2020 to June 2021.

We identified 12 key dates as turning points for mood scores during this period. Figure 2 shows the general trend in the monthly sentiment changes of microblog users during the COVID-19 pandemic. In general, the overall classification results show obvious clustering according to the time series.

In the first stage (July 2020 to October 2020), strong infectious tendencies and unknown virus characteristics exacerbated public panic, and user sentiment ratings were generally low. In the second phase (November 2020 to February 2021), changes in sentiment scores were relatively stable, except for a large decrease in January 2021 and February 2021, possibly due to material shortages and population movement that accelerated virus transmission. In the third phase (March 2021 to June 2021), the proportion of positive emotions gradually increased thanks to the successful development and widespread distribution of vaccines. The data show that the changes in the number of infected people and the effectiveness of pandemic prevention measures were important factors affecting the change in the polarity of public sentiment.

**Figure 3:**
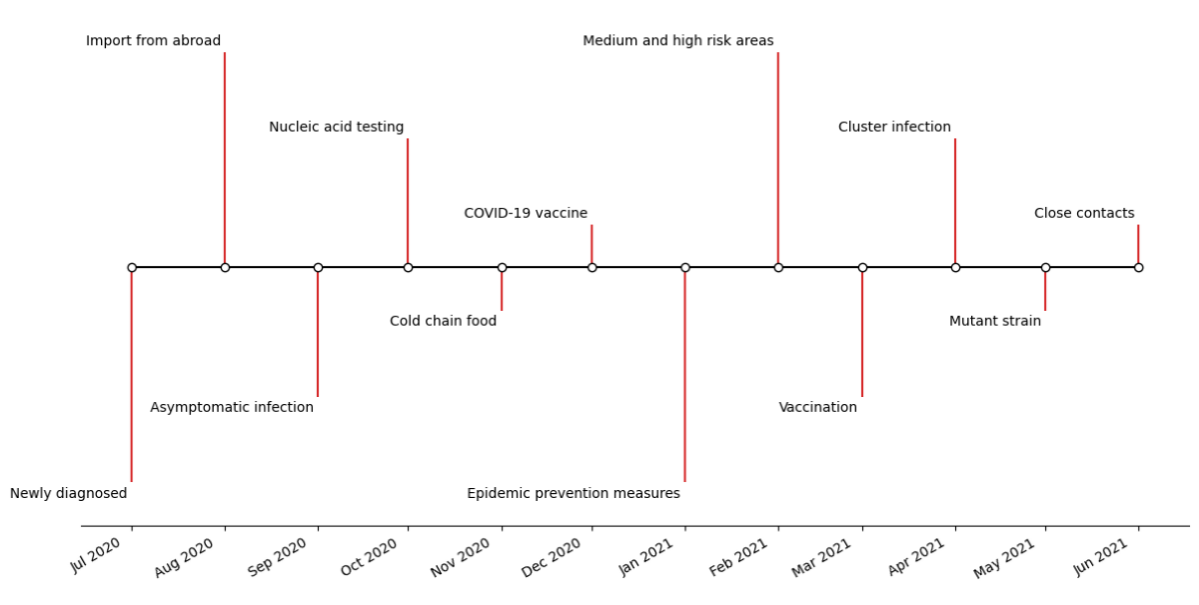
Monthly statistics of the keywords in the hot topic searches regarding COVID-19 pandemic behavior in China on microblogs from July 2020 to June 2021.

In the face of the pandemic, people’s instinctive psychological reaction was panic and anxiety, and these negative emotions were particularly obvious in the first stage. In the second stage, government departments strengthened the control of public opinion on social media. As a result, the public’s confidence about fighting the pandemic was significantly enhanced, and the emotional polarity gradually improved. In the third stage, the proportion of positive emotions increased significantly, and negative emotions gradually turned into affirmation and support for antipandemic actions. Figure 3 shows the keywords in the monthly hot topic searches on the microblogs during the COVID-19 pandemic.

The SnowNLP tool was used to calculate the sentiment value, and the text was classified according to the threshold. The LDA model was then used to mine the topics. The hyperparameters of the model were set as α=50/*K* and γ=0.01. The number of iterations was set at 1000. The number of potential topics was determined as *K*=12 according to the perplexity. The same weight was given to the topics with a monthly cycle by combining the change in the intensity of the influence of the propagation of the microblog information.

We obtained 12 topics from the feature extraction of microblog topics during the COVID-19 pandemic (see Table 5). Among them, pandemic prevention and people’s livelihoods were the focus of public attention.

**Table 5:** Topic feature extraction analysis of microblog comments about the COVID-19 pandemic at different stages in China from July 2020 to June 2021.

Stage and subject number		Subject content	Keywords extracted by the LDA^a^ model
**Phase 1**			
	T1	New confirmed cases	Pandemic rebound, death, persistence, fear, migration, virus spread, contagion risk, pandemic prevention
	T2	Import from abroad	Disinfect, detect, security, risk level, classification, source control, viral activity, personnel contact
	T3	Asymptomatic infection	Isolation, therapy, monitor, incubation personal, close contacts, medical observe, infectiousness, immunity
	T4	Nucleic acid testing (NAT)	24 hours, region, sampling method, screening, antigen detection, sampling point, information input, detection result
**Phase 2**			
	T5	Cold chain food	Traceability, cheek, disinfect, safety, risk, touch, nucleic acid testing, virus
	T6	COVID-19 vaccine	Clinical trials, safety, side effect, output, validity, develop, price, inoculate
	T7	Pandemic prevention measures	Face mask, health, disinfect, body temperature, nucleic acid testing, immunity, quarantine, vaccine
	T8	Medium or high-risk areas	Cases, asymptomatic infection, spread risk, medical insurance, living necessities, regional control, nucleic acid testing, health monitoring
**Phase 3**			
	T9	Vaccination	Vaccination rate, necessity, population immunity, adverse reactions, antibody, allergy, personal protection, inoculation contraindication
	T10	Cluster infection	Source of infection, chain of transmission, close contacts, gathering activity, nucleic acid testing, pandemic prevention awareness, route of transmission, policy control
	T11	Variant strain	Omicron, Delta, critically patients, panic, contagious, pathogenic, mortality rate, drug resistance
	T12	Close contact	Centralized isolation, medical observation, nucleic acid testing, body temperature, abnormal signs, incubation period, clinical symptoms, movement tracking

^a^LDA: latent Dirichlet allocation.

Multimedia Appendix 1 shows the results of topic modeling. In the first stage, the main topics were mainly focused on pandemic prevention and control. In the second stage, in addition to vaccines, food safety became the focus. The core topics of the third stage were COVID-19 vaccination and variants.

### ML-LR Public Opinion Sentiment Analysis With a Sparse Matrix

**Figure 4:**
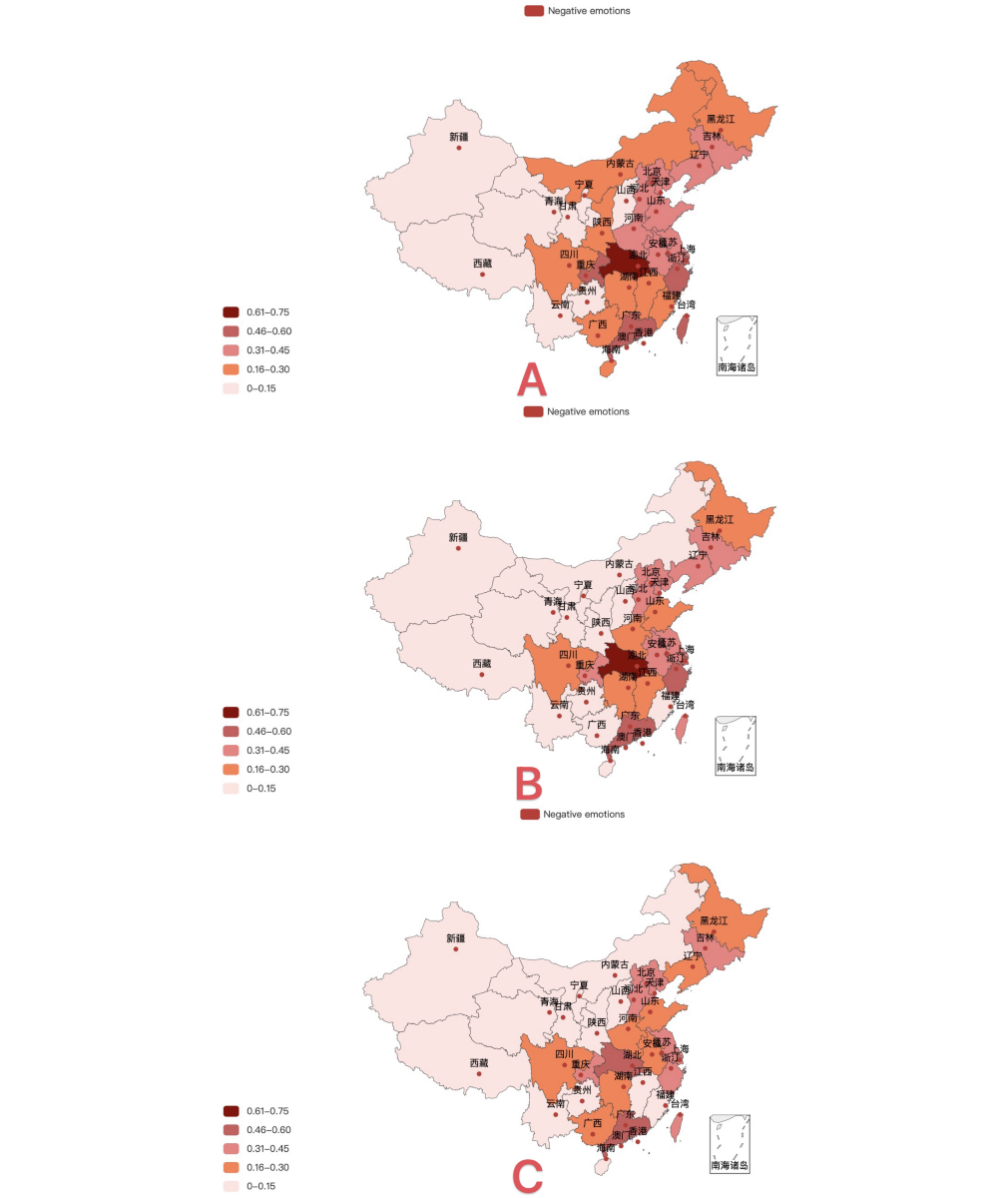
Geographical distribution of posts with negative sentiment by Chinese microblog users from July 2020 to June 2021: (A) phase 1, (B), phase 2, (C) phase 3.

To explore the spatial distribution of public emotions, we subtracted the same types of emotions from provinces and regions in adjacent stages according to regional differences and obtained the spatiotemporal changes in negative emotions, as shown in Figure 4. Taking provincial administrative regions as the basic statistical unit and the 3 evolution stages as fixed time intervals, the natural break point method was used to divide the proportion of negative emotions.

Neighboring provinces had similar emotional characteristics; that is, the spatiotemporal similarity of emotions was high. Moreover, the proportion of negative emotions was related to the severity of the local pandemic, and a high value for negative emotions had a spatial distribution similar to that of areas with a more severe pandemic situation. In addition, provinces with many confirmed cases had a high proportion of negative emotions, while provinces with a small number of confirmed cases had a relatively low proportion of negative emotions.

**Figure 5:**
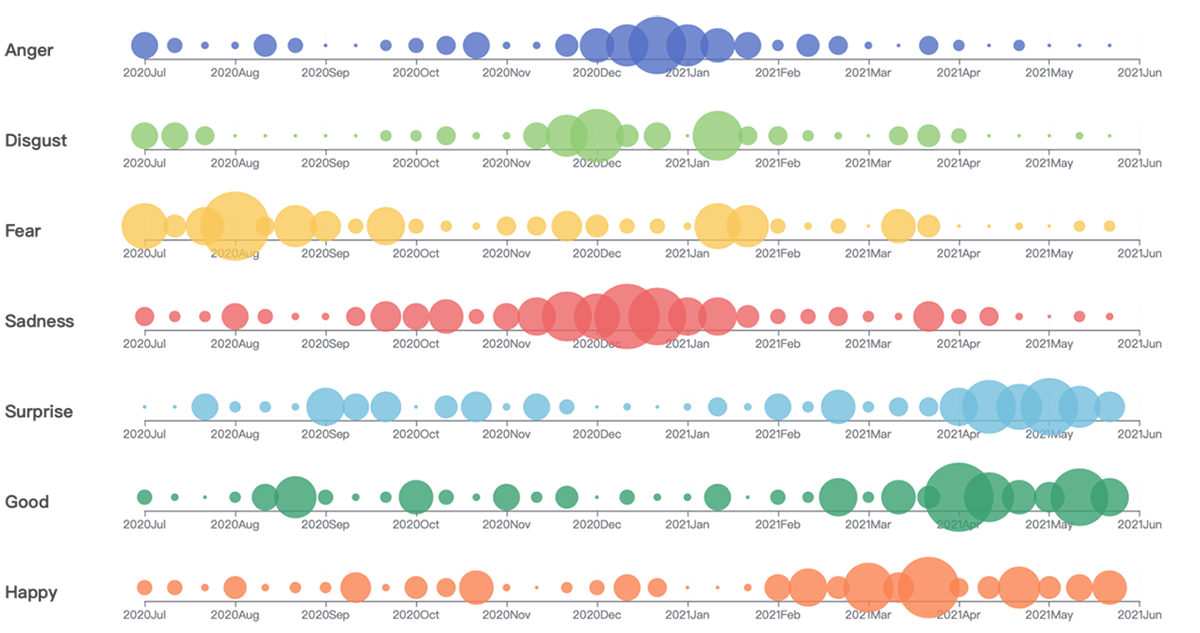
Bubble plot of the temporal trends in 7 sentiments based on microblog comments during the COVID-19 pandemic in China from July 2020 to June 2021.

Combined with China’s daily real-time notifications regarding the pandemic, the first stage of the pandemic in China occurred during the outbreak period, with the number of confirmed cases reaching a peak in August 2020 and the confirmed case rate gradually slowing down since then. As can be seen in Figure 5, during the period from July 2020 to January 2021, anger, disgust, fear, sadness, and other negative emotions prevailed. When combined with topic digging, this result shows that people’s concerns regarding the COVID-19 pandemic included fear of human-to-human transmission, a domestic outbreak, and imported cases from other countries.

During the second stage (February 2021 to June 2021), the sentiment scores basically showed positive sentiment, indicating that the government adopted effective response policies and that public sentiment tended to stabilize again. Positive emotions such as surprise, good, and happy rapidly increased, indicating that the government invested considerable human and material resources at any cost. Therefore, the public was able to observe the determination and effectiveness of the country’s antipandemic efforts. In addition, with the arrival of the inflection point during the third phase of the pandemic, the mass distribution of COVID-19 vaccines increased public confidence that the pandemic could be prevented and controlled and that production, work, and prospects could resume after the pandemic.

To visually present the words occurring at a high frequency for each emotion, we used a word cloud library to generate cloud maps of 70 words for 7 emotions corresponding to positive and negative emotions in the 3 stages, as shown in Table 6. At the same time, high-frequency words were combined with the corresponding current affairs hot spots to explore the differences in and connections of different semantic features in different sentiment classifications.

**Table 6:** The 7 emotional keywords based on COVID-19 pandemic topics found in microblog comment data in China from July 2020 to June 2021.

Topic and keywords			Number of times used
**Topic 1: happy**			
	Sanguine	474	
	Hope	399	
	Happiness	307	
	Relieved	301	
	Believe	268	
	Pray	218	
**Topic 2: good**			
	Health	535	
	Security	490	
	Safety	462	
	Recovery	391	
	Bless	307	
	Great	274	
**Topic 3: surprise**			
	Unforeseen	367	
	Vaccine	350	
	Diagnose	329	
	Outbreak	274	
	Pathogen	241	
	Cure	207	
**Topic 4: sadness**			
	Epidemic	728	
	Collapse	597	
	Hopelessness	582	
	Despair	487	
	Negative	424	
	Chill	278	
**Topic 5: fear**			
	Virus	624	
	Infect	619	
	Danger	481	
	Trepidation	447	
	Horror	410	
	Helpless	368	
**Topic 6: disgust**			
	Rumor	394	
	Oppose	350	
	Keep away	311	
	Shirk	263	
	Back off	231	
	Slackness	196	
**Topic 7: anger**			
	Hurt	368	
	Indignation	359	
	Conceal	301	
	Forbid	248	
	Malice	217	
	Ferocity	145	

In the initial stage of the pandemic, the words occurring at a high frequency included “hopeless,” “danger,” “infect,” and other words as well as “negative,” “rumor,” “helpless,” and many other words that express emotions. In the face of the sudden COVID-19 pandemic, the public was in an extremely unstable emotional state.

In the second stage of the pandemic, the words occurring at a high frequency included “keep away,” “pandemic,” and “pathogen.” Microblog topics focused on orderly prevention and control measures and a joint fight against the pandemic. The positive sentiment of netizens gradually increased, turning from fear to solidarity and cooperation.

In the third stage of the pandemic, words such as “vaccine,” “recovery,” “hope,” and “sanguine” appeared frequently on the microblog. Most people were full of confidence and believed that China could win the battle against the pandemic, expressing positive feelings of positivity, unity, and hope for the future.

### Model Performance Analysis

To verify that the proposed model and method had high accuracy, we calculated accuracy, precision, recall, and the *F*_1_-score, as shown in Equations 12-15:



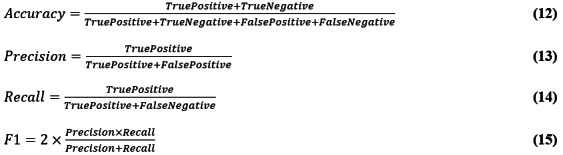



**Figure 6:**
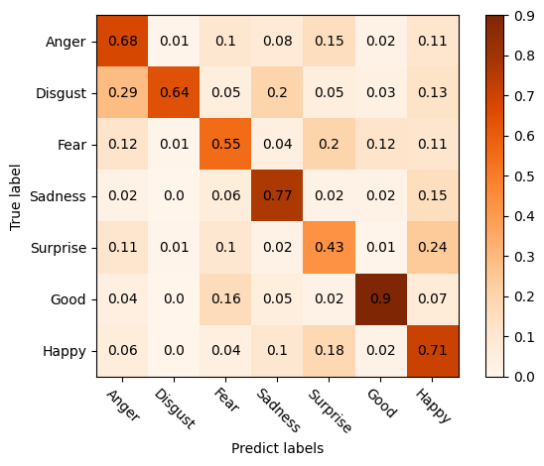
Multiclass confusion matrix for sentiment scoring based on COVID-19 pandemic-related topics in microblog comments in China from July 2020 to June 2021.

The words in the text were converted into a word frequency matrix, and the TF-IDF weight of each word was counted. The TF-IDF matrix was then extracted, and its weight was calculated. Finally, the effectiveness of the model was evaluated by extracting parameters (precision, recall, accuracy) from the confusion matrix, as shown in Figure 6.

First, the word frequency matrix was generated using the text-based data after Chinese word segmentation and data cleaning. The *CountVectorizer* class was called to calculate the word frequency matrix, and the generated matrix was *X*. Second, the *TfidfTransformer* class was called to calculate the TF-IDF value of the term frequency matrix *X*, and the *Weight* matrix was obtained. The *Sklearn* machine learning package was then called to perform the classification operation, the *fit ()* function was called to train, and the predicted class labels were assigned to the *pre* array. Finally, we called the *PCA ()* function of *Sklearn* to reduce these features to 2 dimensions corresponding to the *X* and *Y* axes, from which we could evaluate the algorithm.

Based on the study of the traditional double classification confusion matrix, we divided the confusion matrix into 7 emotions according to the emotional characteristics. Very few data points were misclassified from the matrix by the model. Moreover, the performance of the model in terms of accuracy, recall, and the *F*_1_-score of the weighted average of the 7 emotions “anger,” “disgust,” “fear,” “sad,” “surprise,” “good,” and “happy” was good, as shown in Table 7.

**Table 7:** Performance analysis of a machine learning logistic regression (ML-LR) language processing model fusing a sparse matrix based on Chinese COVID-19 pandemic topics from July 2020 to June 2021.

Measures	Precision	Recall	*F*_1_-score	Support
Negative	0.8447	0.9525	0.9189	175
Positive	0.8158	0.8961	0.8344	428
Accuracy	N/A^a^	N/A	0.8581	603
Macro average	0.8579	0.6138	0.6440	603
Weighted average	0.7982	0.7181	0.7811	603

^a^N/A: not applicable.

## Discussion

### Principal Findings

This study constructed a multidimensional analysis model of public opinion on social media in China during the COVID-19 pandemic from the perspective of spatiotemporal correlations. We proposed an ML-LR model with a sparse matrix to analyze the evolutionary process of public opinion and interpret the dynamic relationship and multidimensional emotional characteristics of public opinion in different spatiotemporal environments. The results show that, due to the different trends in the pandemic situation and prevention and control efforts in different regions, there were differences in the emotional characteristics of public opinion. The amount of public opinion data at many different levels was similar in the temporal and spatial distributions, and the amount of public opinion data was positively correlated with the number of new cases. With the rapid spread of the COVID-19 pandemic, the monthly amount of public opinion data increased. As the COVID-19 pandemic was gradually controlled, the amount of monthly public opinion data showed a downward “zigzag” trend. The spatial distribution of the amount of public opinion data was positively correlated with the distribution of COVID-19 spread, and the provinces with more public opinion data were mostly those areas with a more serious COVID-19 situation. This study can provide theoretical support and a practical reference for government and public health safety departments to deal with public health emergencies.

### Comparison With Prior Work

In traditional research of public opinion on social media, the topic model method is often combined with a text clustering algorithm [12,23,25], but this approach is limited by the problem of insufficiently labeled data, making it difficult to reflect the changes in public mood. This paper proposes an ML-LR model with a fusion coefficient matrix to overcome these problems. In addition, social media data embedded with geographical location information provides valuable evaluation indicators for the study of public opinion characteristics, but the time span and geographical location range selected by traditional research of public opinion on social media are of limited size or are not representative [4,17]. This study captures the dynamic changes in public sentiment characteristics through multidimensional, spatiotemporal analysis of public opinion.

### Limitations and Future Work

We will conduct further research regarding 2 aspects in the future. On one hand, when analyzing public sentiment during a pandemic, user comments may come from multiple social media platforms [25,26]. Subsequent research will consider adding data from other social media platforms, strive to include a more comprehensive user group, and describe more appropriate regional characteristics of public opinion. On the other hand, considering that the spread of the pandemic has certain geospatial heterogeneity under the joint influence of geographical proximity, transportation network, and pandemic prevention measures, taking provincial administrative regions as geospatial measurement units has certain limitations [27-29].

In future research, we will discuss how to obtain a multiscale visual analysis unit of the pandemic and public opinion according to the superposition of the transmission mode of the COVID-19 pandemic and multiscale geographic space. We will also mine the process of propagation during the pandemic from a more granular scale in terms of time and space.

### Conclusions

The analytic method of public opinion on social media proposed in this study can effectively reflect the characteristics of public opinion in different regions and different periods of time. It can also provide theoretical support and a practical reference for the analysis of public opinion in major public health events, as well as provide correct guidance for government departments and effective control of the propagation of network public opinion.

## Appendix

Multimedia Appendix 1 Visual analysis of the COVID-19 topic modeling results based on microblog comments from July 2020 to June 2021 in China.

## Abbreviations

API: application programming interface
BERT: Bidirectional Encoder Representations from Transformers
BTM: biterm topic model
LDA: latent Dirichlet allocation
ML-LR: machine learning logistic regression
TF-IDF: term frequency-inverse document frequency

## Data Availability

The data sets containing confirmed COVID-19 cases and microblog comments are publicly available on the official websites of the Chinese Center for Disease Control and Prevention and the Sina microblog.

The coded data and filtered files are made available on request from the corresponding author.

## References

[1] Zhuang M, Li Y, Tan X, Xing L, Lu X (2021). Analysis of public opinion evolution of COVID-19 based on LDA-ARMA hybrid model. Complex Intell Systems.

[2] Priyadarshini I, Mohanty P, Kumar R, Sharma R, Puri V, Singh PK (2022). A study on the sentiments and psychology of twitter users during COVID-19 lockdown period. Multimed Tools Appl.

[3] Lopez CE, Gallemore C (2021). An augmented multilingual Twitter dataset for studying the COVID-19 infodemic. Soc Netw Anal Min.

[4] Zhu B, Zheng X, Liu H, Li J, Wang P (2020). Analysis of spatiotemporal characteristics of big data on social media sentiment with COVID-19 epidemic topics. Chaos Solitons Fractals.

[5] Lu Z (2022). Analysis model of college students' mental health based on online community topic mining and emotion analysis in novel coronavirus epidemic situation. Front Public Health.

[6] Ogbuokiri B, Ahmadi A, Bragazzi NL, Movahedi Nia Z, Mellado B, Wu J, Orbinski J, Asgary A, Kong J (2022). Public sentiments toward COVID-19 vaccines in South African cities: An analysis of Twitter posts. Front Public Health.

[7] Ahmad W, Wang B, Xu H, Xu M, Zeng Z (2021). Topics, sentiments, and emotions triggered by COVID-19-related tweets from Iran and Turkey official news agencies. SN Comput Sci.

[8] Sun K, Wang H, Zhang J (2022). The impact factors of social media users' forwarding behavior of COVID-19 vaccine topic: Based on empirical analysis of Chinese Weibo users. Front Public Health.

[9] Albahli S (2022). Twitter sentiment analysis: An Arabic text mining approach based on COVID-19. Front Public Health.

[10] Sarirete A (2021). A bibliometric analysis of COVID-19 vaccines and sentiment analysis. Procedia Comput Sci.

[11] Elsaka T, Afyouni I, Hashem I, Al Aghbari Z (2022). Spatio-temporal sentiment mining of COVID-19 Arabic social media. IJGI.

[12] Johnson AK, Bhaumik R, Nandi D, Roy A, Mehta SD (2022). Sexually transmitted disease-related Reddit posts during the COVID-19 pandemic: latent Dirichlet allocation analysis. J Med Internet Res.

[13] Box-Steffensmeier JM, Moses L (2021). Meaningful messaging: Sentiment in elite social media communication with the public on the COVID-19 pandemic. Sci Adv.

[14] Liu J, Liu L, Tu Y, Li S, Li Z (2022). Multi-stage Internet public opinion risk grading analysis of public health emergencies: An empirical study on Microblog in COVID-19. Inf Process Manag.

[15] Liu S, Liu J (2021). Public attitudes toward COVID-19 vaccines on English-language Twitter: A sentiment analysis. Vaccine.

[16] Yousefinaghani S, Dara R, Mubareka S, Papadopoulos A, Sharif S (2021). An analysis of COVID-19 vaccine sentiments and opinions on Twitter. Int J Infect Dis.

[17] Hu T, Wang S, Luo W, Zhang M, Huang X, Yan Y, Liu R, Ly K, Kacker V, She B, Li Z (2021). Revealing public opinion towards COVID-19 vaccines with Twitter data in the United States: spatiotemporal perspective. J Med Internet Res.

[18] Zhang X, Yang Q, Albaradei S, Lyu X, Alamro H, Salhi A, Ma C, Alshehri M, Jaber II, Tifratene F, Wang W, Gojobori T, Duarte CM, Gao X (2021). Rise and fall of the global conversation and shifting sentiments during the COVID-19 pandemic. Humanit Soc Sci Commun.

[19] Jang H, Rempel E, Roth D, Carenini G, Janjua NZ (2021). Tracking COVID-19 discourse on Twitter in North America: infodemiology study using topic modeling and aspect-based sentiment analysis. J Med Internet Res.

[20] Storey VC, O'Leary DE (2022). Text analysis of evolving emotions and sentiments in COVID-19 Twitter communication. Cognit Comput.

[21] Alhashmi SM, Khedr AM, Arif I, El Bannany M (2021). Using a hybrid-classification method to analyze Twitter data during critical events. IEEE Access.

[22] Gao H, Guo D, Wu J, Li L (2022). Weibo users' emotion and sentiment orientation in traditional Chinese medicine (TCM) during the COVID-19 pandemic. Disaster Med Public Health Prep.

[23] Xie R, Chu SKW, Chiu DKW, Wang Y (2021). Exploring public response to COVID-19 on Weibo with LDA topic modeling and sentiment analysis. Data Inf Manag.

[24] Xie Q, Zhang X, Ding Y, Song M (2020). Monolingual and multilingual topic analysis using LDA and BERT embeddings. Journal of Informetrics.

[25] Ghasiya P, Okamura K (2021). Investigating COVID-19 news across four nations: a topic modeling and sentiment analysis approach. IEEE Access.

[26] Hou K, Hou T, Cai L (2021). Public attention about COVID-19 on social media: An investigation based on data mining and text analysis. Pers Individ Dif.

[27] Rahman MM, Khan NI, Sarker IH, Ahmed M, Islam MN (2022). Leveraging machine learning to analyze sentiment from COVID-19 tweets: A global perspective. Eng Rep.

[28] Naseem U, Razzak I, Khushi M, Eklund PW, Kim J (2021). COVIDSenti: a large-scale benchmark Twitter data set for COVID-19 sentiment analysis. IEEE Trans. Comput. Soc. Syst.

[29] Guo F, Zhou W, Lu Q, Zhang C (2022). Path extension similarity link prediction method based on matrix algebra in directed networks. Computer Communications.

