# Correction: Public Opinion About COVID-19 on a Microblog Platform in China: Topic Modeling and Multidimensional Sentiment Analysis of Social Media

**DOI:** 10.2196/57233

**Published:** 2024-02-14

**Authors:** Feipeng Guo, Zixiang Liu, Qibei Lu, Shaobo Ji, Chen Zhang

**Affiliations:** 1 Modern Business Research Center Zhejiang Gongshang University Hangzhou China; 2 School of Management and E-Business Zhejiang Gongshang University Hangzhou China; 3 School of International Business Zhejiang International Studies University Hangzhou China; 4 Sprott School of Business Carleton University Ottawa, ON Canada; 5 General Manager's Office Hangzhou Gaojin Technology Co, Ltd Hangzhou China

In “Public Opinion About COVID-19 on a Microblog Platform in China: Topic Modeling and Multidimensional Sentiment Analysis of Social Media” (J Med Internet Res 2024;26:e47508) the authors noted one error.

In the “Methods” section of the Abstract, the following sentence:

Finally, a machine learning linear regression (ML-LR) model combined with a sparse matrix was proposed to explore the evolutionary trend in public opinion on social media and verify the high accuracy of the model.

Has been changed to read as follows:

Finally, a machine learning logistic regression (ML-LR) model combined with a sparse matrix was proposed to explore the evolutionary trend in public opinion on social media and verify the high accuracy of the model.

The correction will appear in the online version of the paper on the JMIR Publications website on February 14, 2024 together with the publication of this correction notice. Because this was made after submission to PubMed, PubMed Central, and other full-text repositories, the corrected article has also been resubmitted to those repositories.

