# Supplementary figures and images for: Public Opinion About COVID-19 on a Microblog Platform in China: Topic Modeling and Multidimensional Sentiment Analysis of Social Media
Source: J Med Internet Res. 2024 Jan 31;26:e47508. doi: 10.2196/47508 (PMC10833090; doi:10.2196/47508)

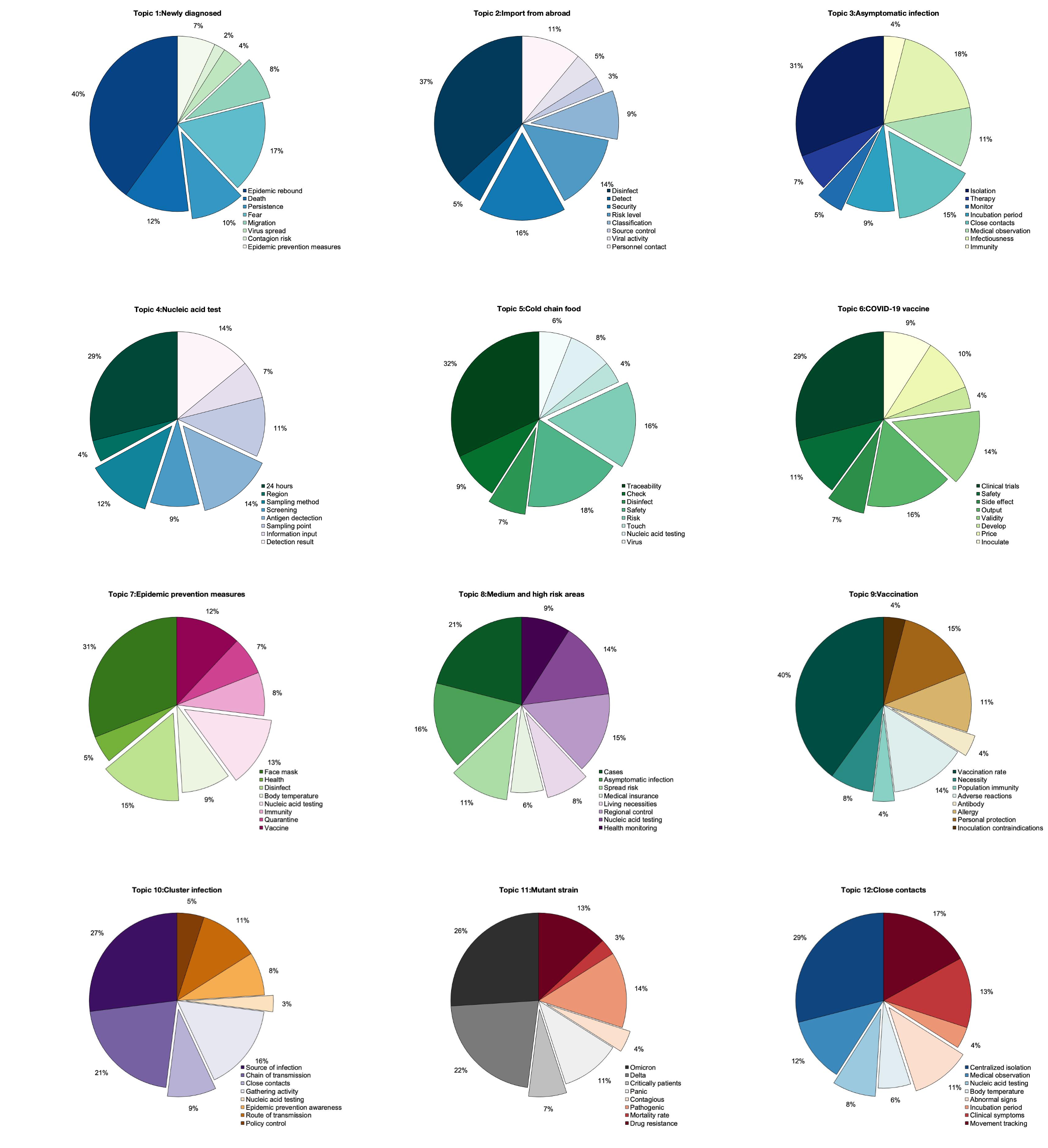

Supplement: Multimedia Appendix 1 [file jmir_v26i1e47508_app1.png]
